# Surgical technique for mitral valve repair in dogs using a novel method to anchor artificial chordae tendineae with emphasis on key intraoperative decision points

**DOI:** 10.3389/fvets.2024.1444742

**Published:** 2024-12-18

**Authors:** Noriko Isayama, Takeshi Mizuno, Sayaka Suzuki, Kenta Sasaki, Erika Maeda, Yusuke Uchimura

**Affiliations:** ^1^Department of Cardiology, Uenonomori Animal Hospital, Tokyo, Japan; ^2^Tokyo Animal Cardiothoracic Surgery (TACTS), Tokyo, Japan; ^3^Veterinary Medical Center, Graduate School of Agricultural and Life Sciences, The University of Tokyo, Tokyo, Japan; ^4^Japan Small Animal Medical Center, Saitama, Japan

**Keywords:** canine, mitral insufficiency, mitral valve repair, surgical technique, surgical management

## Abstract

**Objectives:**

Surgical options for mitral valve repair in dogs are unstandardized and influenced by various factors. This study describes a four-point surgical technique (MI-4) to reduce mitral regurgitation and provides data from a study of dogs with 12 months of follow up.

**Methods:**

Twenty-five dogs with stages C or D mitral insufficiency were treated by one of two surgeons using the MI-4 procedure at Ueno no Mori Animal Hospital between October 2021 and May 2023. The surgical technique comprised: (I) determination of the valve annulus dimensions by measuring between the trigones, (II) triad-anchored chordae tendinea reconstruction, (III) determination of the appropriate position and number of chordae tendineae on the leaflets, and (IV) appropriate height determination. The regurgitation percentage was measured using B-mode color Doppler flow in the atrium in the four-chamber left long-axis view.

**Results:**

There were no intraoperative complications, and 23, 23, and 18 dogs were successfully re-evaluated at 1, 6, and 12 months, respectively (5 dogs have not yet reached the 12-month follow-up point). The regurgitation percentage decreased from 73.0% (interquartile range, 58.1–81.5%) preoperatively to 2.1% (0.0–8.8%), 4.6% (0.1–10.8), and 1.3% (0.0–7.1) at 1, 6, and 12 months postoperatively, respectively. All surviving dogs improved clinically.

**Conclusion:**

The MI-4 surgical technique was performed in dogs with mitral valve insufficiency with no significant complications. The surgery reduced the regurgitation percentage postoperatively, with benefits seen at least 12 months after surgery.

## 1 Introduction

Mitral insufficiency is a potentially fatal ailment in dogs that can lead to heart failure. In the QUEST randomized study evaluating two treatments for dogs with myxomatous degenerative mitral valve disease, the overall median survival following the onset of heart failure was 188 days (1).

The American College of Veterinary Internal Medicine consensus guidelines were established to aid in the treatment of dogs with myxomatous mitral valve disease; however, surgical intervention is not widely used in current clinical practice (2). This is partly owing to the complexity of surgical procedures and the variability in outcomes; for example, in a study of 18 dogs with mitral regurgitation repaired surgically, six patients did not survive surgery and, in the remainder, the period of freedom from congestive heart failure ranged from 4 months to 3 years (3). Experienced surgical teams can adopt several strategies and techniques to improve surgical outcomes, including cardiopulmonary bypass control and nuances in surgical techniques. However, these are often not standardized and are frequently facility specific. The valves and tendon cords in dogs with mitral insufficiency resemble those in humans with Barlow’s disease (4) and may rupture in multiple locations. Valve replacement surgery is not a practical option because an appropriately safe and reliable anticoagulation protocol has not been established in dogs. In addition, many of the techniques used in humans, such as removal of the papillary muscle and folding of tendon chords, are not used in dogs because of differences in the size of the heart and lack of reports of experience with such techniques. Therefore, it is impossible to apply most of the principles of techniques used in human cardiac surgery to dogs.

These factors contribute to the difficulties in performing cardiac surgery in dogs with myxomatous mitral valve disease and, together with the association identified between duration of cardiac arrest during surgery and outcomes (3), highlight the need to develop a mitral valve repair technique that can minimize or eliminate these challenges.

A four-point surgical technique (MI-4) was developed to simplify mitral valve repair and ensure consistent and effective repair. In this study, we describe the MI-4 technique and report the 12-month follow-up results.

## 2 Materials and methods

### 2.1 Animals

The present study included a cohort of 25 client-owned dogs diagnosed with mitral insufficiency due to myxomatous degenerative mitral valve disease and treated with MI-4 at the Ueno no Mori Animal Hospital between October 2021 and May 2023. Of the 25 dogs, 14 were diagnosed with myxomatous mitral valve disease stage C (current or past clinical signs of heart failure) disease, whereas 11 were diagnosed with stage D (clinical signs of failure refractory to standard treatment for stage C heart failure) disease (2). The study protocol was reviewed and approved by the Institutional Review Board of the Ueno no Mori Animal Hospital (approval number: 230111). All the owners of the included dogs provided informed consent for the procedure. The surgical procedure was performed by one of two surgeons; one surgeon had 2 years of experience and performed 18 surgeries, whereas the other had 4 years of experience and performed 7 surgeries.

### 2.2 Anesthesia and cardiopulmonary bypass

General anesthesia was administered during the surgical procedure as previously described (5), and each dog was placed in the right lateral recumbency position following which an incision was made in the neck for cannulation. Intravenous heparin (300 U/kg) was administered, and the activated clotting time was measured to be >250 s using the i-STAT ACT Kaolin test kit (Abbot Japan, Tokyo, Japan). A venous drainage cannula (Flexmate; Toyobo, Shiga, Japan) was placed in the left external jugular vein, and a return cannula (DLP Cardiopulmonary Bypass Cannula; Medtronic, Minneapolis, MN, United States) was inserted into the common carotid artery. The dog was then connected to an oxygenator and circuit (MERA-HP-Exelung TPC; Senko Medical Instrument, Tokyo, Japan), and cardiopulmonary bypass was established using the MERA-Extracorporeal Circulation System TRUSYS (Senko Medical Instrument). A left fifth thoracotomy was performed, and the pericardium was incised parallel to the phrenic nerve. A root cannula was inserted into the aorta (Ao) to administer cardioplegia. Once the flow rate of the cardiopulmonary bypass stabilized, a modified del Nido cardioplegia solution (20 mL/kg) was administered for open-heart surgery.

### 2.3 Surgical technique

**Figure 1:**
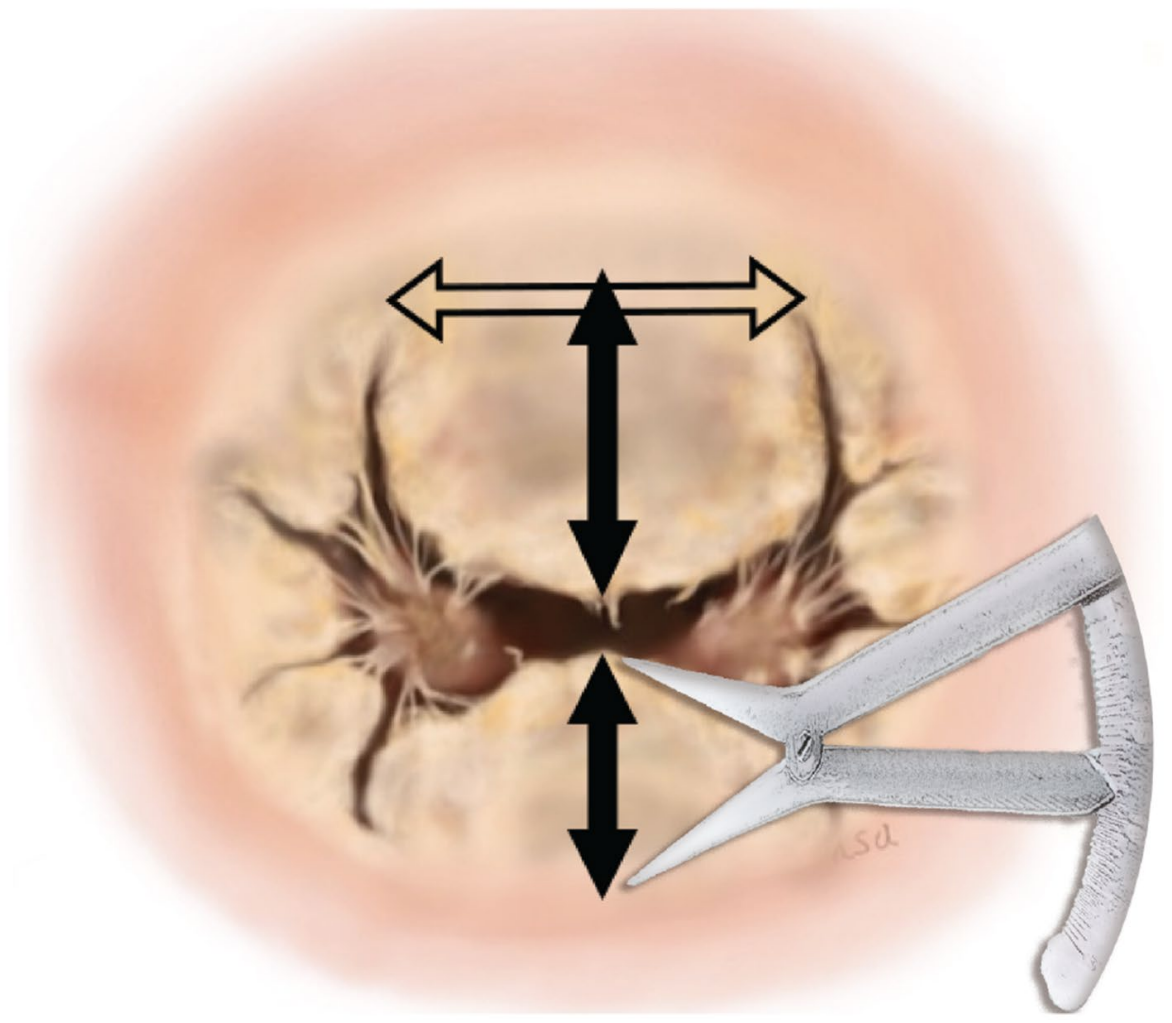
Measurement of annular dimensions. The horizontal arrow represents the measurement of the annular diameter between the trigones of the mitral valve leaflet. The top vertical arrow represents the measurement of the anterior leaflet. The bottom vertical arrow represents the measurement of the posterior leaflet.

MI-4 comprises four key technical points. The first is determining the valve annular dimension by measuring the anterior leaflet. The technique was consistent in approach through measurement of the annular diameter between the trigones of the mitral valve leaflet and to use the same distance between the trigones as the annular diameter (Figure 1). The measurements proceed as follows. First, the distance between the trigones should be measured, followed by measurement of the lengths of the anterior (A2) and posterior (P2) mitral valves. The annular diameter should be approximately the same length as the distance between the trigones (6). However, it is important to note that measurements using a caliper can lead to errors of ~1 mm. Such measurement errors can make a considerable difference in small-breed dogs and, to ensure the size is adequate, 60–65% of the combined lengths of A2 + P2 was calculated to determine whether the determined annulus size would be adequate for the valve. If the determined annulus size is not within 60–65% of the combined A2 + P2 lengths, we recommend remeasurement of the length in between the trigones. After estimating the annular size, the annulus was sutured using the modified DeVega technique from trigone to trigone (7), and a sizer (custom-made non-commercial acryl tube) was inserted to reduce the annulus to the target size. The technique of final confirmation of the valve size is the same as that described in humans (8) and, if the sizer does not extrude from the anterior leaflet, the size is considered acceptable.

**Figure 2:**
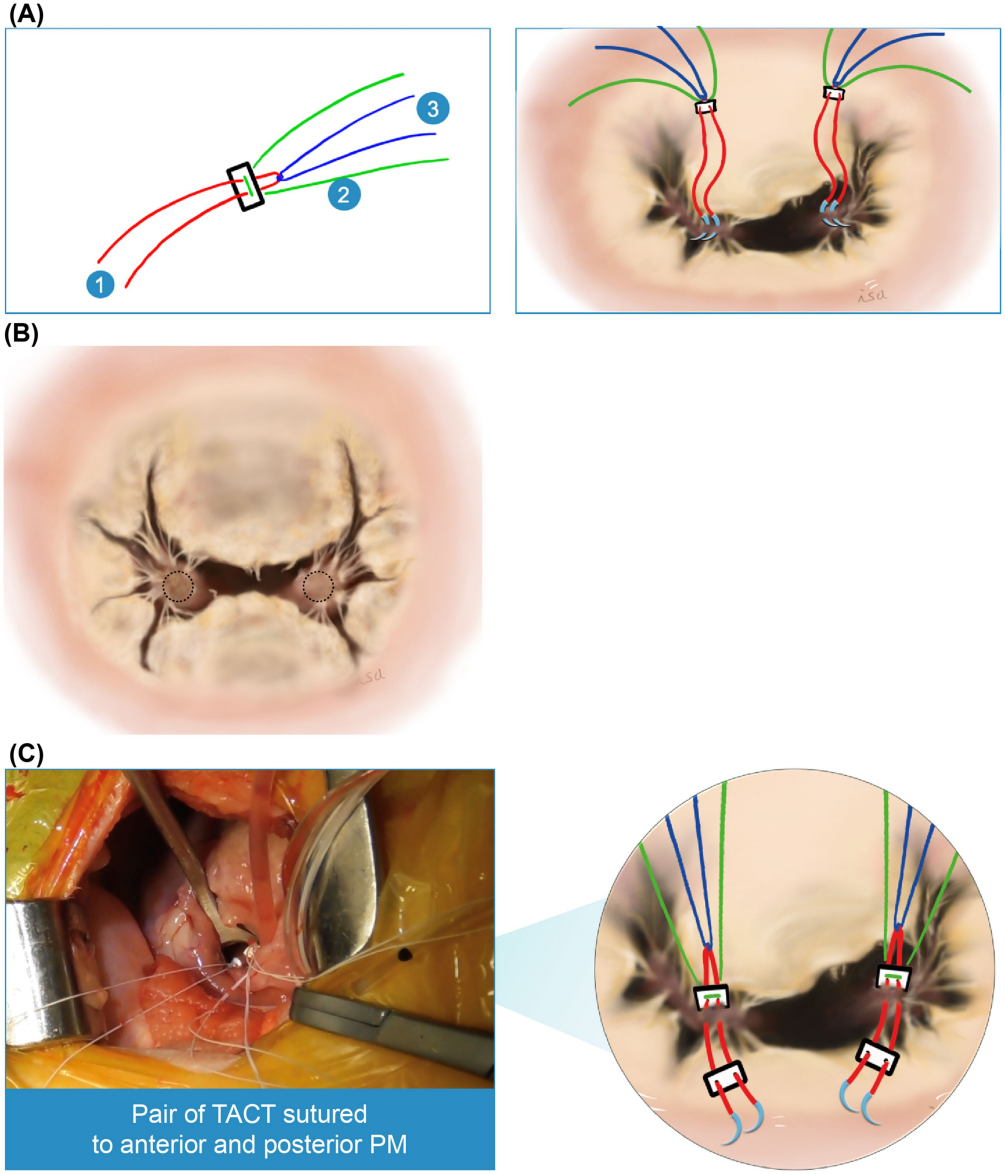
Triad-anchored chordae tendinea reconstruction method. **(A)** Two pledgets (Pledget E, Matsuda Ika Kogyo, Tokyo, Japan) are used, each comprising a complex of three Gore-Tex sutures (labeled 1–3) (Gore-Tex CV-6, W.L. Gore & Associates, Newark, DE, United States). Accordingly, only two attachments are required for each papillary muscle. **(B)** The sutures should be positioned on the fibrous tissue of each papillary muscle, avoiding interference with the native chordae tendineae to ensure proper movement. There is typically an unobstructed area below the chordae tendineae, extending to the anterior leaflet (dotted circle), which is the preferred suture location. **(C)** Illustration of a pair of triad-anchored chordae tendineae (TACT) sutured to the anterior and posterior papillary muscles (PPM).

Second, a triad-anchored chordae tendinea reconstruction method was established. This method involves a complex of three Gore-Tex sutures and two Gore-Tex patches, requiring only two attachments to each papillary muscle (PPM) (Figure 2A). Two sutures were inserted through the PPM. The sutures should be placed on the fibrous tissue-covered area of each papillary muscle, avoiding any interference with the native chordae tendineae to ensure proper movement. Typically, there is an unobstructed area below the chordae tendineae, extending to the anterior leaflet, making it a suitable location for suturing (Figure 2B). This results in “sandwiching” of the muscle with the Gore-Tex patch, and the sutures are tied down, thereby providing three pairs of chordae from one PPM (Figure 2C).

**Figure 3:**
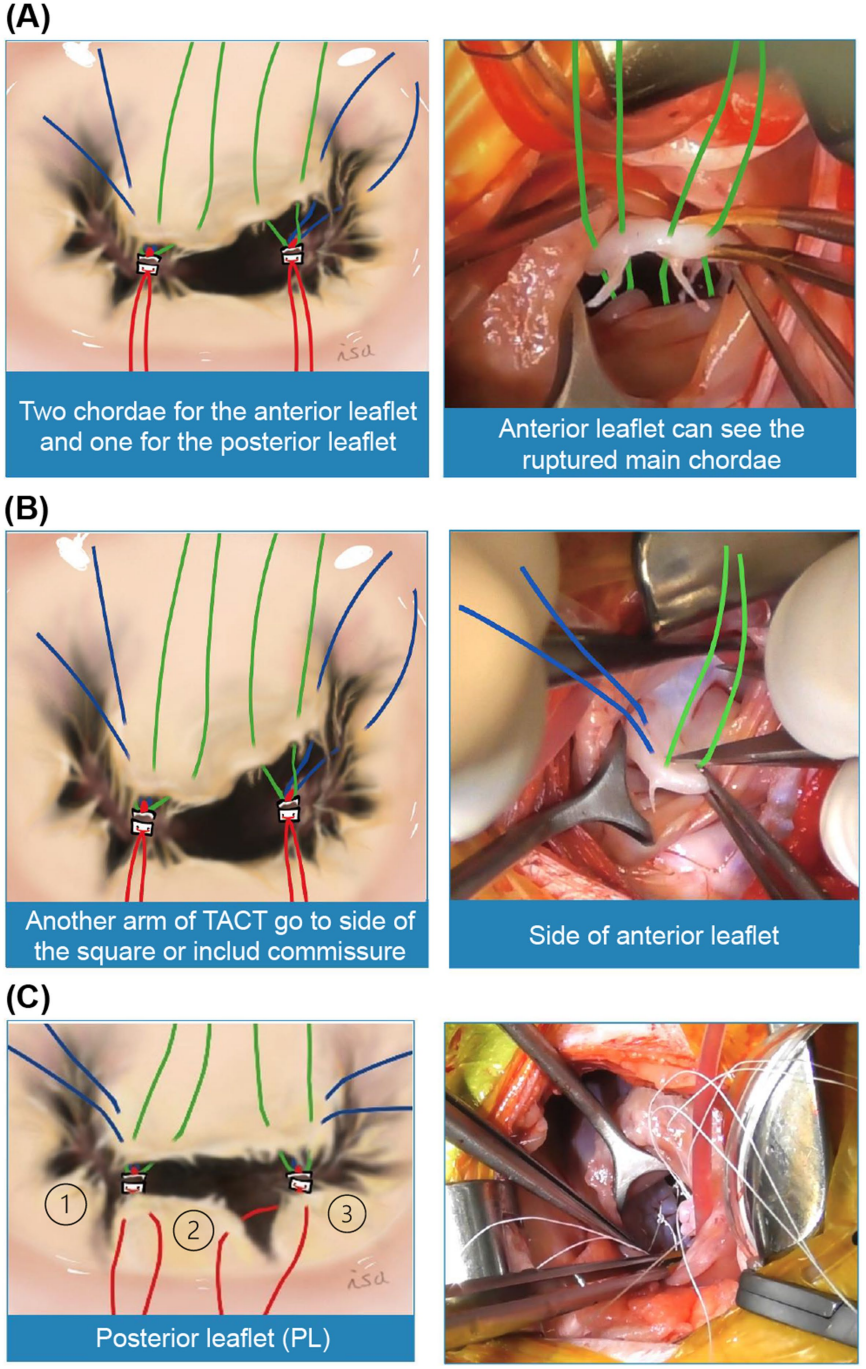
Reconstruction of the six chordae tendineae. **(A)** We propose using two pairs of chords from each triad-anchored chordae tendineae (TACT) on each papillary muscle (PPM) for the anterior leaflet. **(B)** The other two chordae can be tied to both sides of the square. A leakage test should be performed before placing the sutures. If severe prolapse is detected in the commissure, one arm on the side can include a commissure valve to create a single valve. If the valve has clefts that cannot be included between the sutures, a magic suture can be made. After this key point is completed, the next area of focus is the posterior leaflet. **(C)** Subsequently, the posterior leaflets (PLs) can be divided into three sections: P1, P2, and P3. Among these, P2 is the largest and is generally rectangular. In our previously reported procedure, a pair of TACT from each PPM is used to create new posterior chordae tendineae. Each suture arm from the TACT is placed at the corner of the P2 rectangle. If P2 is wide and has numerous native chordae tendineae, the other suture arm can be used to sandwich and reinforce the tendineae. Another suture can be placed if P1 or P3 shows prolapse. Therefore, mitral valve defects can be corrected.

Third, six chordae tendineae were typically reconstructed using a pair of triad-anchored chordae tendineae sutured to each anterior and posterior PPM (Figure 3A). Two chordae from each triad-anchored chordae tendineae sutured to the anterior and posterior PPMs were used for the anterior leaflet, whereas one was used for the posterior leaflet. The anterior leaflet is shaped like a square or wide oval and sometimes has a cleft. Considering a square shape, the model cases had two main chordae tendineae on the square’s right and left base corners, which were the sides of the coaptation zone with the posterior leaflet. The native main chordae can be thick tendons, a group of several thin tendons, or ruptured, with only one bump remaining. The anterior leaflet was sutured through both sides of the native main chordae, and the arms on top of the native main chordae, which may become elongated or weakened in the future, were tied. However, if the native main chordae were not visible, the square’s base was trisected, and the other arm was placed at one-third of the length of the base from the right and left corners. The other two chordae for the anterior leaflet were tied to both sides of the square (Figure 3B). If the leak test indicated leakage from the commissure, one arm on the side could include a commissure valve to create a single valve. If there were clefts that could not be included between the sutures, a magic suture (8) was made using a 6–0 monofilament non-absorbable suture. The posterior leaflet was divided into three parts: P1, P2, and P3 (Figure 3C). P2 was the largest, rectangular, and the main component of the posterior leaflet. In the surgical procedure, a pair of triad-anchored chordae tendineae originating from the anterior and posterior PPMs was used to create new posterior chordae tendineae. Each arm of the suture was placed on each corner of the rectangular P2 using each triad-anchored chordae tendineae from the PPM. If P2 is wide and contains several native chordae tendineae, the other arm of the suture may be used to sandwich and reinforce the tendineae. In the case of any leakage identified in P1 or P3, another arm of the suture might be placed to fix the issue.

Fourth, accurately determining the height of the triad-anchored chordae tendineae is crucial for preventing regurgitation during mitral valve reconstruction. Knots of the triad-anchored chordae tendineae were tied at the level of the mitral valve annulus to ensure proper coaptation. To control regurgitation, the heights of the triad-anchored chordae tendineae were matched when there was no obvious leakage during the leak test, as even small discrepancies could affect the length of the coaptation zone. Notably, the chordae from each PPM should have the same height.

In summary, the MI-4 method for mitral repair in dogs consists of four key phases: (1) determination of valve annular dimensions based on the distance between the trigones, cross-referenced to 60–65% the combined length of S2 and P2; (2) triad-anchored chordae tendinea reconstruction; (3) establishment of an appropriate position and number of chordae tendineae on the leaflets; and (4) determination of the appropriate height and consistency of knots.

### 2.4 Postoperative follow up

The percentage of regurgitation was measured by calculating the maximal ratio of the regurgitant jet area signal to the left atrial area (RJA/LA area) using B-mode color Doppler flow in the atrium in the four-chamber left long-axis view. Routine echocardiography, including the left atrium/Ao ratio (LA:Ao), normalized left ventricular internal dimensions (LVIDDN), early mitral valve diastolic filling velocity (E-wave velocity), late atrial systolic filling velocity (A-wave velocity), and mean pressure gradient (mean PG), was performed preoperatively as well as 1, 6, and 12 months postoperatively.

### 2.5 Statistical analyses

The normality of the data distribution was examined using the Shapiro–Wilk test, and depending on the distribution of the data, either parametric or non-parametric tests were employed for further analysis. Preoperative and 1-month post-surgery data were compared based on the stage of the valve disease using Wilcoxon signed-rank tests with continuity correction. Comparisons incorporating the preoperative and 1-, 6-, and 12-month postoperative data on LA:Ao, LVIDDN, E- and A-wave velocities, mean PG, RJA/LA area, and medication dosages were performed using Friedman tests. Statistical analyses were performed using EZR software (version 1.55; Division of Hematology, Saitama Medical Center, Jichi Medical University, Saitama, Japan) (9). Statistical significance was set at *p* < 0.05.

## 3 Results

The sample population comprised 9 Chihuahuas, 3 Toy Poodles, 3 Cavalier King Charles Spaniels, 3 Malteses, 1 Miniature Schnauzer, 1 Shih Tzu, and 5 mixed-breed dogs. The median body weight of the dogs was 3.7 kg (interquartile range [IQR], 3.1–5.4 kg), and the median age was 11.0 years (IQR, 9.8–11.7 years). There were no intraoperative complications, and 23 dogs were discharged. One dog died of pancreatitis 6 days postoperatively and another dog died 2 days postoperatively due to protracted seizures. The remaining 23 dogs were successfully reevaluated at 1 and 6 months, with 18 of these evaluated 12-months postoperatively (the remaining 5 dogs are alive but have not reached the 12-month follow-up point yet). All surviving dogs showed clinical improvement.

### 3.1 Echocardiographic examination

**Figure 4:**
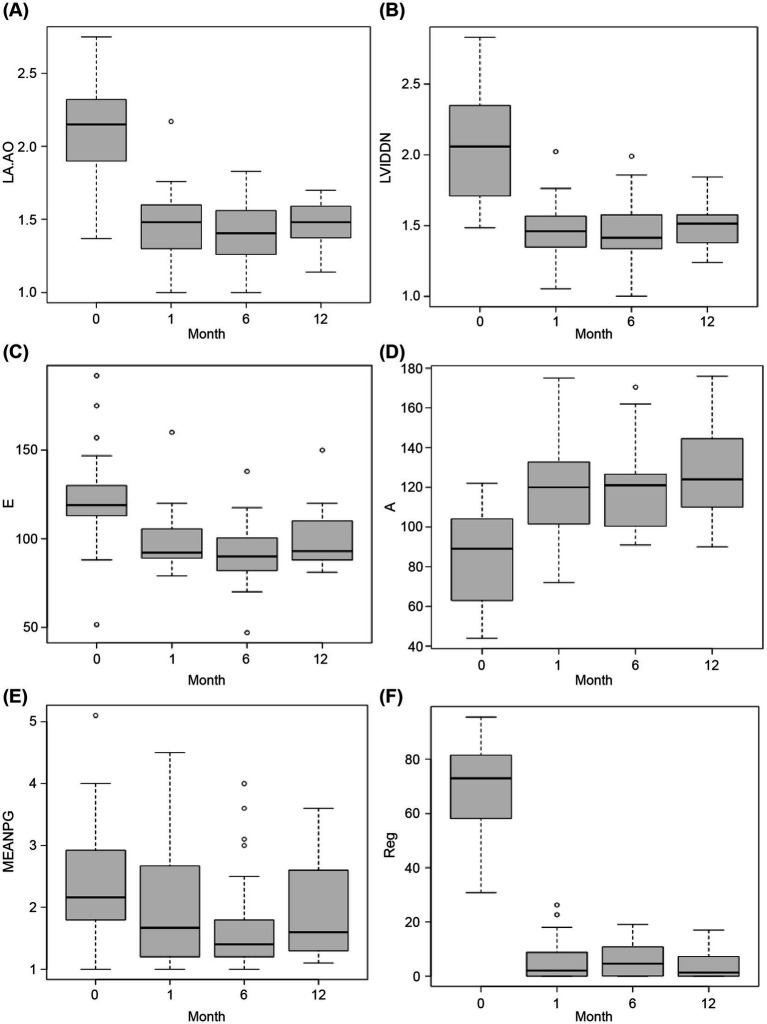
Comparison of pre-and postoperative data. The x-axes (labeled “Month”) indicate the timepoint as follows: 0, preoperatively; 1, 1-month postoperatively; 6, 6-months postoperatively; and 12, 12-months postoperatively. Statistical comparison of preoperative and postoperative values was performed using the Wilcoxon signed-rank test with continuity correction. **(A)** Left atrium/aorta ratio (LA:Ao) **(B)** Normalized left ventricular internal dimensions (LVIDDN) **(C)** Early mitral valve diastolic filling velocity (E-wave; in cm/s) **(D)** Late atrial systolic filling velocity (A-wave; in cm/s) **(E)** Mean pressure gradient (MEAN PG; in mmHg) **(F)** Regurgitant jet area/left atrial area (RJA/LA area; the y-axis, labeled Reg, represents the percentage regurgitation).

There was a significant decrease in the LA:Ao and LVIDDN 1-month postoperatively compared with the preoperative values (LA:Ao preoperatively: 2.15 [IQR 1.9–2.3] vs. 1 month postoperatively: 1.51 [IQR 1.4–1.6], *p* < 0.001; LVIDDN preoperatively: 2.06 [IQR 1.7–2.4] vs. 1 month postoperatively: 1.43 [IQR 1.3–1.5], *p* < 0.001; Figures 4A,B); a further reduction was observed in LA:Ao (6 months postoperatively: 1.39 [IQR 1.3–1.6], *p* < 0.01) and LVIDDN (6 months postoperatively: 1.40 [IQR 1.3–1.5], *p* < 0.0001) 6 months postoperatively. Values were non-significantly higher at 12 months relative to 6 months for both LA:Ao (1.48 [IQR 1.4–1.6]) and LVIDDN (1.51 [IQR 1.4–1.6]), but both still remained significantly lower than preoperatively (LA:Ao *p* < 0.01; LVIDDN *p* < 0.01). There was a non-significant decrease in E-wave and a significant increase in A-wave values 1 month postoperatively (E-wave preoperatively: 118.9 cm/s [IQR, 113.0–130.0] vs. 1 month postoperatively: 92.0 cm/s [IQR 89.0–105.5], *p* = 0.081; A-wave preoperatively: 89.0 cm/s [IQR 63.0–104.0] vs. 1 month postoperatively: 120.0 cm/s [IQR 101.5–132.7], *p* = 0.0072; Figures 4C,D). These changes were maintained 6 months postoperatively for both the E-wave (6 months postoperatively: 90.0 cm/s [IQR 82.0–100.5], *p* = 0.040) and A-wave (6 months postoperatively: 121.0 cm/s [IQR 100.5–126.5], *p* < 0.01) values, with the E-wave values at 6 months being significantly lower than the preoperative values, unlike the 1-month values. However, at 12 months, the E-wave values increased to slightly higher than the 1-month values and were non-significantly different to the preoperative values (12 months postoperatively: 93.0 cm/s [IQR 88.0–110.0], *p* = 0.382). The A wave values, however, increased further and became more significantly higher than the preoperative values (12 months postoperatively: 124.0 cm/s [IQR 110.0–139.5], *p* < 0.001).

Relative to the preoperative values, there was no significant change in mean PG 1 month (preoperatively: 2.2 mmHg [IQR 1.8–2.9] vs. postoperatively: 1.67 mmHg [IQR 1.2–2.7], *p* = 1.00), 6 months (6 months postoperatively: 1.4 mmHg [IQR 1.2–1.8], *p* = 0.076), or 12 months postoperatively (12 months postoperatively: 1.6 mmHg [IQR 1.3–2.5], *p* = 0.225).

### 3.2 Regurgitant jet area/left atrial area

A significant reduction in the percentage of regurgitation was observed 1 month postoperatively compared with the preoperative percentage (preoperatively: 73.0% [IQR 58.1–81.5] vs. postoperatively: 2.1% [IQR 0.0–8.8], *p* < 0.001; Figures 4E,F). A slight increase in the percentage of regurgitation was observed 6 months postoperatively; however, this remained significantly lower than the preoperative value (6 months postoperatively: 4.6% [IQR 0.1–10.8], *p* < 0.001), resulting from a decrease in LA area due to remodeling. The percentage of regurgitation decreased again at the 12-month point, remaining significantly lower than the preoperative value (12 months postoperatively: 1.3% [IQR 0.0–7.1], *p* < 0.001). The RJA did not change.

### 3.3 Disease stage differences

There was no significant difference in the average operative duration between dogs with stage C and dogs with stage D valve disease (Stage C: 185.0 min [IQR 175.0–200.0] vs. Stage D: 238.0 min [IQR 205.0–257.5], *p* = 0.0884), but dogs with stage C disease had a significantly larger reduction in RJA/LA area at 1 month postoperatively (Stage C: 100.0 [IQR 97.6–100.0] vs. Stage D: 88.5 [IQR 82.5–94.3], *p* = 0.00425).

### 3.4 Medication use

At 1-month postoperatively, there was a significant reduction in the median dose of pimobendan (preoperatively: 1.6 mg/kg/day [IQR 1.3–2.0] vs. postoperatively: 0.0 mg/kg/day [IQR 0.0–0.2], *p* < 0.0001), amlodipine (preoperatively: 0.4 mg/kg/day [IQR 0.3–0.5] vs. postoperatively: 0.0 mg/kg/day [IQR 0.0–0.0], *p* < 0.01), furosemide (preoperatively: 1.9 mg/kg/day [IQR 0.0–5.7] vs. postoperatively: 0.0 mg/kg/day [IQR 0.0–0.0], *p* = 0.018), and benazepril (preoperatively: 0.6 [IQR 0.0–1.4] vs. postoperatively: 0.0 mg/kg/day [IQR 0.0–0.0], *p* = 0.015), as well as a non-significant reduction in torasemide (preoperatively: 0.2 mg/kg/day [IQR 0.0–0.5] vs. postoperatively: 0.0 mg/kg/day [IQR 0.0–0.0], *p* = 0.055), compared with the preoperative values. These changes were maintained or improved at 12 months postoperatively compared to preoperatively (pimobendan, 0.0 mg/kg/day [IQR 0.0–0.0], *p* < 0.0001; amlodipine, 0.0 mg/kg/day [IQR 0.0–0.2], *p* < 0.01; furosemide, 0.0 mg/kg/day [IQR 0.0–0.0], *p* = 0.015; benazepril, 0.0 mg/kg/day [IQR 0.0–0.0], *p* = 0.031; torasemide, 0.0 mg/kg/day [IQR 0.0–0.0], *p* = 0.055).

## 4 Discussion

This study described the use of a four-point surgical technique (MI-4) to control mitral regurgitation, with accompanying data from 23 dogs of various breeds. The techniques were developed in the hope that they could be used by other surgeons. Preliminary data indicate that these techniques were highly effective at reducing mitral valve regurgitation in the hands of two different surgeons. Overall, clinical improvement in mitral regurgitation was achieved in all dogs 1 month postoperatively, with no occurrence of intraoperative complications. The changes were largely maintained 6 and 12 months after surgery. Notably, there was no significant change in the mean PG relative to the preoperative values. The use of several medications was reduced significantly 1 month postoperatively, and this reduction was maintained at 6 and 12 months postoperatively. However, our data should be considered preliminary, and as indicated below, further studies are required to confirm our promising findings.

Notably, although there are variations in the reported outcome measures among studies and the lengths of follow up, our results are in line with those of previous studies. A previous study of 18 dogs found a significant reduction in the LA:Ao ratio at 6 months following mitral valve repair performed using various surgical techniques (3). In that study, only 12 of the 18 dogs survived the surgery, with the causes of death including a tear in the atrioventricular groove (*n* = 1), disseminated systemic thrombosis (*n* = 1), and low-output heart failure (*n* = 4). Notably, all 18 dogs in that study had established severe mitral regurgitation and congestive heart failure preoperatively. Another study of 55 dogs identified significant reductions in LVIDDN, LA:Ao, and E-wave values up to 2 years following mitral valve repair performed using the loop-in-loop technique; in that study, 50 of the dogs survived to discharge, and all medications for mitral valve disease were stopped 3 months postoperatively (10). Of the five dogs that died in the study, one developed an interventricular septal hematoma immediately following surgery, one developed a sudden neurological deficit 3 h following wean from general anesthesia, one died of thrombosis following the development of atrial fibrillation, another died from hypotension due to heart failure following the development of atrial fibrillation, and one died from disseminated intravascular coagulation. In another study, significant reductions in LA:Ao up to 3 months following mitral valve repair were reported in 48 small-breed dogs who underwent implant of expanded polytetrafluoroethylene chordal prostheses (5). In that study, 45 of 48 dogs survived to discharge and a decrease in medications prescribed to the dogs at 1 month postoperatively was also reported; furthermore, by 6 months postoperatively, 29 of the evaluated dogs were medication free (5). The causes of death in those that did not survive to discharge were bleeding during recovery from anesthesia (*n* = 1) and thrombosis 5 (*n* = 1) and 8 (*n* = 1) days following surgery.

Medical and surgical options exist for managing mitral valve regurgitation (2); however, medical treatment is currently associated with poor outcomes (1), and surgical options are not widely available because of the complexity of the procedures and heterogeneity in outcomes. These challenges highlight the need for a consistent surgical approach that achieves consistent outcomes.

Our proposed MI-4 approach comprises four key points. The first involves determination of the valve annular dimension by measuring the anterior leaflet. Commercial equipment is available to determine the annular size using the trigones of the mitral valve as landmarks in humans; however, such equipment is unsuitable for small-breed dogs. Measurement of the dimensions of the aortic valve or left ventricular outflow tract has been attempted to determine the size of the mitral valve (11); however, we could not prove the utility of this approach, finding that the resulting size estimation is too small and results in mitral stenosis. Indeed, a paper reporting on long-term outcomes in dogs undergoing mitral valve repair with this technique does not describe the detailed reasons for the development of mitral stenosis in the dogs studied (12). In our study, the annular size was estimated based on a trigonal-based sizing method previously described in humans (6), which can be applied to dogs of various sizes and has been shown to avoid the development of functional mitral stenosis and systolic anterior motion. A smaller annulus size can contribute to the development of systolic anterior motion of the mitral valve (13) and a longer coaptation zone, and an excessively long coaptation zone can cause systolic anterior motion of the mitral valve (14, 15). An excessively short annular diameter can also lead to mitral stenosis. Conversely, an excessively large annular diameter may lead to failure of regurgitation prevention. The second key point involves the establishment of a triad-anchored chordae tendineae. Note that other surgical teams perform mitral valve repair in a different order to us, performing chordae tendinea reconstruction before the annulus suturing, although that is strictly the surgeon’s preference and is not expected to influence the outcome of surgery. Regarding the chordae tendinea reconstruction technique, we believe that our approach of securing three chords into the papillary muscle with one suture has several advantages. First, our technique reduces the number of approaches required for PPM and also has the advantage of reducing the risk of rupture of the atrium, helping obtain a clear view deep in the left ventricle. In addition, this method is likely to be faster than approaching the PPM multiple times, a concept also key to the loop-in-loop technique (10), as it involves suturing three pairs of chords at once instead of one pair of chords three times separately, although no formal speed comparison has been performed. A potential disadvantage of our approach is that if the first chord ruptures, unfortunately all three will collapse [also a limitation of the loop-in-loop technique (10)]. In the third key point, six chordae tendineae are typically reconstructed using a pair of triad-anchored chordae tendineae sutured to each anterior and posterior PPM. The final key point of the technique involves accurately determining the height of the triad-anchored chordae tendineae. The knots of the TACT are tied at the level of the mitral valve annulus. In cases of deeply tied knots, the valve leaflet cannot retain blood within the ventricle. Therefore, leakage occurs, and the leak test fails. In contrast, if the knots are tied too high, prolapse of the valve leaflets may occur. In addition, to ensure regurgitation control, the heights of the knots should be identical on the anterior/posterior leaflets. Notably, the chordae from each PPM must have the same height (not the same length). It is essential to maintain uniformity in the heights of all knots; this is considered by the authors to be critical for the technical success of the procedure, as miniscule discrepancies can affect the length of the coaptation zone and surgical outcomes, and it is our belief that most cases of severe regurgitation after mitral valve surgery result from height problems.

The main limitations of this study were the relatively small number of study participants (*n* = 25). Further studies with larger populations of participants are warranted to validate our findings. In addition, although the provision of the MI4 surgical technique aims to improve the consistency of the surgical technique for surgery to treat myxomatous degenerative mitral valve disease, there remains an unavoidable element of subjectivity to some aspects of the approach that rely on the surgeon’s own judgment. Nevertheless, we feel that the proposed technique offers an important protocol to improve consistency in the surgical approach to this technically challenging procedure.

## 5 Conclusion

In this study, the MI-4 surgical technique reduced the number of intraoperative decision points and may have led to effective control of regurgitation as well as to the prevention of intraoperative complications. The MI-4 technique effectively decreased the heart size without causing mitral stenosis. The results from this study have the potential to guide the establishment of a standard technique for mitral valve repair surgery. Further studies with larger numbers of dogs and surgeons are warranted.

## Data Availability

The raw data supporting the conclusions of this article will be made available by the authors, without undue reservation.
